# Neuroscience and Brain Death Controversies: The Elephant in the Room

**DOI:** 10.1007/s10943-018-0654-7

**Published:** 2018-06-21

**Authors:** Joseph L. Verheijde, Mohamed Y. Rady, Michael Potts

**Affiliations:** 1 Department of Physical Medicine and Rehabilitation, Mayo Clinic, 13400 E Shea Blvd, Scottsdale, AZ 85259 USA; 2 Department of Critical Care Medicine, Mayo Clinic Hospital, 5777 E Mayo Blvd, Phoenix, AZ 85054 USA; 3 Department of Philosophy and Religion, Methodist University, 5400 Ramsey Street, Fayetteville, NC 28311-1498 USA

**Keywords:** Brain death, Ethics, Neuroscience, Organ donation, Religion

## Abstract

The conception and the determination of brain death continue to raise scientific, legal, philosophical, and religious controversies. While both the President’s Commission for the Study of Ethical Problems in Medicine and Biomedical and Behavioral Research in 1981 and the President’s Council on Bioethics in 2008 committed to a biological definition of death as the basis for the whole-brain death criteria, contemporary neuroscientific findings augment the concerns about the validity of this biological definition. Neuroscientific evidentiary findings, however, have not yet permeated discussions about brain death. These findings have critical relevance (scientifically, medically, legally, morally, and religiously) because they indicate that some core assumptions about brain death are demonstrably incorrect, while others lack sufficient evidential support. If behavioral unresponsiveness does not equate to unconsciousness, then the philosophical underpinning of the definition based on loss of capacity for consciousness as well as the criteria, and tests in brain death determination are incongruent with empirical evidence. Thus, the primary claim that brain death equates to biological death has then been de facto falsified. This conclusion has profound philosophical, religious, and legal implications that should compel respective authorities to (1) reassess the philosophical rationale for the definition of death, (2) initiate a critical reappraisal of the presumed alignment of brain death with the theological definition of death in Abrahamic faith traditions, and (3) enact new legislation ratifying religious exemption to death determination by neurologic criteria.

## Introduction

The debate on the validity of the concept of brain death, its determination in clinical practice, and the philosophical, legal, and religious controversies it has raised not only remains unabated but also has increased in intensity. Brain death was introduced as human death on the assumption of its equivalency with biological death. Over the past 50 years, empirical knowledge has proven that this assumption is false. In this article, we outline the historical background of brain death and discuss how conceptual revisions have silently transformed brain death from a biological definition of human death to a non-biological one. We summarize empirical knowledge from contemporary neuroscience challenging the validity of death determination with neurologic criteria. The philosophical, legal, and religious implications of a faulty death determination in clinical practice are discussed. We further emphasize that Abrahamic faith traditions’ (Judaism, Christianity, and Islam) acceptability of the concept brain death was conditioned upon: (1) equivalency with biological death, (2) clinical determination with scientifically verifiable criteria and tests, and (3) alignment with the theological definition of death, i.e., the separation of the soul from the human body. Brain death determination fails to meet these conditions, which raises serious moral questions for organ donation practice. Therefore, new legislation should be enacted ratifying religious exemption to death determination by neurologic criteria.

## Historical Background

The Ad Hoc Committee of the Harvard Medical School to Examine the Definition of Brain Death (1968) published its report introducing the definition of death by neurologic criteria or “brain death.” The primary purpose of the Ad Hoc Harvard Committee was “to define irreversible coma as a new criterion for death.” However, disputes about the concept’s validity broke out almost immediately. The Harvard Committee (1968) stated, without providing further substantiating evidence, that the validity of brain death criteria was grounded in empirical knowledge after exclusion of reversible causes. The Harvard Committee determined “the characteristics of a *permanently* (emphasis in the original) nonfunctioning brain” to be (1) unreceptivity and unresponsiveness, (2) absent movements or breathing, and (3) absent reflexes. An electroencephalogram, if available, should be used as a confirmatory test. Its general statement that any organ “that no longer functions and has no possibility of functioning again is for all practical purposes dead” is unmistakably true. However, introducing a new definition of death requires proponents to formulate a philosophically coherent underlying concept; in this case, the notion that the irreversible and complete dysfunction of a single organ (i.e., the brain) can indeed constitute the biological death of the organism as a whole. Then, a set of physiologic criteria must be identified showing congruence with the definition and a regimen of tests must be in place demonstrating that the conditions contained in the criteria have materialized. These steps have been outlined in the three-part framework of the definition-criteria-tests model as proposed by Bernat et al. (1981). Initially, human death was conceptually understood as the cessation of the integrated functioning of the organism as a whole (Bernat et al. 1981; The President’s Commission 1981). It was later replaced by the organism’s inability to commerce with the external environment as demonstrated by the absence of spontaneous breathing, which is to be considered the organism’s fundamental drive to exist (The President’s Council on Bioethics 2008). The current definition of neurologic death is the irreversible cessation of all functions of the brain, including the brainstem, and is reflected in the legal definition of death enacted in the Uniform Determination of Death Act (UDDA) (National Conference of Commissioners on Uniform State Laws 1981). The criterion of death, i.e., “the general measurable condition that satisfies the definition of death being both necessary and sufficient for death” (Bernat 2006) is the whole-brain formulation. The tests must reliably confirm the manifestation and the irreversible nature of the cessation of all functions of the entire brain in a patient. “The selection of the tests to prove that the criterion is satisfied is solely a medical matter” (Bernat et al. 1981). Therefore, accepted medical standards apply solely to the tests employed and not to the UDDA criteria of death.

The scientific legitimacy of the definition of brain death depends on the availability of empirical evidence, demonstrating (1) the validity of the new set of characteristics of a nonfunctioning brain, and (2) confirmation that diagnostic tests have a sufficiently high level of sensitivity and specificity. In fact, the process of determining death must be error-proof. “Every test or diagnostic criterion in medicine has a trade-off between sensitivity and specificity, but the diagnosis of death is unique in that it demands 100% specificity and 0% chance of false-positive error” (Shewmon 2017). As Nair-Collins and Miller have pointed out, proposing a new theory of organismic death “should [further] be appraised on the basis of the usual theoretical virtues such as coherence with other well-accepted theories, unification of disparate phenomena under an overarching conceptual or ontological framework, and simplicity; and it should be shown to be superior to the older view in these regards” (Nair-Collins and Miller 2017). They posited that if a convincing rationale of each of these elements is absent, the proposed new theory should be rejected.

With a philosophical rationale for the concept of brain death being absent at that time, the President’s Commission (PC) for the Study of Ethical Problems in Medicine and Biomedical and Behavioral Research in 1981 confirmed commitment to a biological definition of death and formulated the “definition of death by neurological criteria.” It argued that “[o]ne characteristic of living things which is absent in the dead is the body’s capacity to organize and regulate itself. In animals, the neural apparatus is the dominant locus of these functions. In higher animals and man, regulation of both maintenance of the internal environment (homeostasis) and interaction with the external environment occurs primarily within the cranium” (The President’s Commission 1981 p. 32). Therefore, the PC posited that “death is that moment at which the body’s physiological system ceases to constitute an integrated whole.” It then adopted the characteristics of a nonfunctioning brain (i.e., unresponsiveness, apnea, and absent brainstem reflexes) proposed by the Ad Hoc Harvard Committee and added that “medically accepted tests must be used to confirm the diagnosis of brain death” (The President’s Commission, 1981 pp. 30–33). As a result, the PC implicitly adjudicated the brain to be the complex organizer and regulator of bodily functions. The PC further clarified that “[p]hysicians… now know what evidence is needed to attest the cessation of brain functions to be complete and irreversible,” implicitly assigning the authority for death determination to medicine (The President’s Commission 1981 p. 46).

The PC preferred a biological definition of death over a non-biological one such as the higher brain death definition (Veatch 1975) or a definition based on a “concept of personal identity” (Green and Wikler 1980). In this way, it underscored the weight it placed on the role of science in defining death. Biological and non-biological definitions differ in the ways their respective validity is being assessed. Within scientific theory, non-biological definitions are commonly characterized as hypothetical constructs, i.e., they cannot be proven or disproven by empirical methods. In contrast, biological definitions must be supported by empirical evidence. For example, if death is defined biologically as the “death of the organism as a whole,” then given appropriate criteria and testing, this view can be empirically tested. However, if death is defined as “permanent loss of personal identity,” the concept of “personal identity” must be fleshed out philosophically (and there is no philosophical unanimity on the nature of personal identity). A further step must also be taken to determine which parts of the brain and/or body must be absent to insure the loss of personal identity.

Finally, the PC expressed “that legislatures ought to set the rules for determining human death and that those rules should recognize brain-oriented techniques of establishing death because traditional standards often cannot be employed with patients whose respiration and circulation are artificially maintained” (The President’s Commission 1981 p. 55). The PC argued that “[t]he statute, ought not to reinforce the misimpression that there are different ‘kinds’ of death, defined for different purposes, and hence that some people are ‘more dead’ than others” (The President’s Commission 1981 p. 60). That same year, the UDDA was drafted and approved by the American Medical Association and the American Bar Association (National Conference of Commissioners on Uniform State Laws 1981). The statute defines that “an individual who has sustained either (1) irreversible cessation of circulatory and respiratory functions, or (2) irreversible cessation of all functions of the entire brain, including the brain stem, is dead. A determination of death must be made in accordance with accepted medical standards.” Of note here is that the UDDA does not specify a medical standard or tests for diagnosing brain death (Yanke et al. 2016).

## Conceptual Revisions

It is fair to say that the peer-reviewed literature contains an extensive number of medical publications challenging the premise that brain death equates to the biological death of a human being (Byrne and Weaver 2004; Joffe 2007; Karakatsanis 2008; Potts et al. 2000; Shewmon 1998, 2012; Zamperetti et al. 2004). In response to the mounting criticism and to avoid further erosion of the brain death definition, the President’s Council on Bioethics (PCB) agreed to review the existing rationale for brain death. The PCB published its White Paper, *Controversies in the Determination of Death*, and recognized that the “[c]linical and pathophysiological facts about the ‘whole-brain death’ condition are better understood today than they were in 1968 or 1981” (The President’s Council on Bioethics 2008 p. 7). Accepting this time-induced knowledge gap, the PCB set out to re-examine “the clinical and pathophysiological facts that call the neurological standard into question” (The President’s Council on Bioethics 2008 p. 58). Although the PCB acknowledged that sufficient evidence had emerged to conclude that in brain dead patients “some of the body’s parts continue to work together in an integrative way,” it maintained that the concept of brain death is still valid (The President’s Council on Bioethics 2008 p. 60). To justify its position, the PCB abandoned the central tenet that death determination is made on the basis of the cessation of integrated functioning of the organism as a whole, and with it the “false assumptions that the brain is the ‘integrator’ of vital functions” (The President’s Council on Bioethics 2008 p. 60). The PCB then offered a new theory of life and death based on what they called “the vital work of the organism.” “… [A] living organism engages in self-sustaining, need-driven activities critical to and constitutive of its commerce with the surrounding world. These activities are authentic signs of active and ongoing life. When these signs are absent, and these activities have ceased, then a judgment that the organism as a whole has died can be made with confidence” (The President’s Council on Bioethics 2008 pp. 90–91). The PCB postulated that the absence of spontaneous breathing in an unconscious patient uniquely proves the failure of the organism to commerce with the surrounding world, although they grant that “… even in a patient with total brain failure, some of the body’s parts continue to work together in an integrated way for some time—for example, to fight infection, heal wounds, and maintain temperature. If these kinds of integration were sufficient to identify the presence of a living ‘organism as a whole,’ total brain failure could not serve as a criterion for organismic death, and the neurological standard enshrined in law would not be philosophically well-grounded” (The President’s Council on Bioethics 2008 p. 60). Without further justification, the PCB selected the absence of spontaneous breathing as the unique identifier of death in an unconscious person. Shewmon (2009) countered that the inner drive to breathe is also absent in conscious patients with lower brainstem lesions, thus challenging the notion that an inner drive to breathe is a necessary feature of organismic wholeness. He further raised the question of why other immanent work on a holistic level should not also count (Shewmon 2009). To date, such fundamental disagreements have not been resolved.

The PCB correctly anticipated that clinical and pathophysiologic facts pertaining to brain death would continue to emerge. However, some of these facts, though readily available in peer-reviewed medical journals, appear to have not yet fully permeated the debate on the validity of brain death. In the years following the PCB’s White Paper, neuroscience has added new knowledge about the functioning of the brain and its potential for recovery after brain injury. Thus far, neurogenesis (Boldrini et al. 2018; Ernst et al. 2014; Gonçalves et al. 2016; Gross 2000) and neuroplasticity (Huang et al. 2017; Sharma et al. 2013) have been described in the adult brain. Both phenomena are potentially paradigm changing in the understanding of recovery of consciousness in the severely injured human brain. Considering these scientific advances, Farisco and Evers (2017) posited that “the traditional way of depicting the unconscious as a dimension deeply separated from and even opposed to consciousness is misleading and overly simplistic.”

## Neuroscience: The Elephant in the Room

**Fig. 1:**
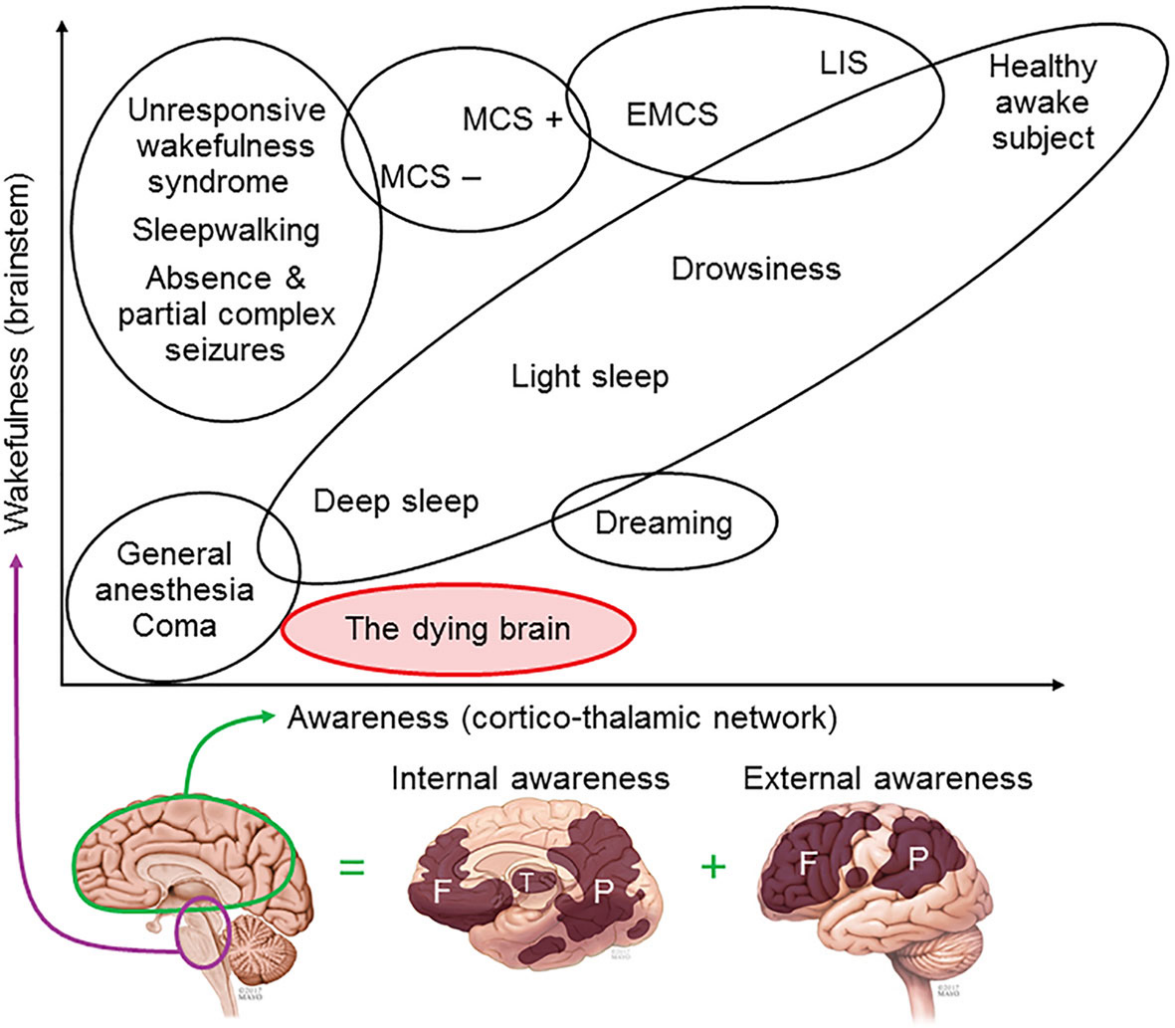
The Phenotypic spectrum of disorders of consciousness. Consciousness consists of wakefulness (arousal), internal awareness (of the self), and external awareness (of the external environment) (Laureys 2005). The diagram schematically illustrates functional areas related to internal and external awareness (telencephalon and diencephalon) and wakefulness (mesencephalon and rhombencephalon) and their interrelationships under different physiologic, pharmacologic, and pathologic alterations of consciousness (Di Perri et al. 2014). Neuroscience research also suggests that latent and covert awareness without wakefulness may be present in the dying brain (Parnia et al. 2014; Rady and Verheijde 2016; Rouleau et al. 2016). Bedside behavioral assessment of unresponsiveness does not necessarily equate with absent capacity for consciousness. *LIS* locked-in-syndrome, *MCS* minimally conscious state, *EMCS* emergence from MCS. (Reproduced and modified from [(Di Perri et al. 2014; Rady and Verheijde 2016)] with the permission of the publisher, Elsevier)

The original conception of brain death was driven by the clinical observation of irreversible coma or unconsciousness in severely brain-injured patients who were dependent on mechanical ventilation. The cessation of capacity for consciousness has underpinned the equation of brain death with human death in the USA, Canada, and the UK, as well as in other international practice guidelines (Shemie et al. 2006, 2014; The Academy of Medical Royal Colleges 2008; The President’s Council on Bioethics 2008; Wijdicks et al. 2010). There have been major advances in the field of neuroscience research in human consciousness since the inception of the term “brain death” 50 years ago (Beecher and Ad Hoc Committee of the Harvard Medical School to Examine the Definition of Brain death 1968). Rapid advances in neuroimaging, electrophysiology, and metabolic studies have improved the understanding of the functional neuroanatomy and neurophysiology of the human brain and consciousness (Fig. 1) (Di Perri et al. 2014; Laureys 2005; Peterson et al. 2015; Rady and Verheijde 2016). These advances have also detected both latent and covert conscious awareness in patients with severe brain injuries who, following a neurologic clinical examination, were determined to lack consciousness (Naci et al. 2017; Sinai et al. 2017). The term “coma dépassé,” or deep coma (Mollaret and Goulon 1959), used almost 60 years ago is now considered meaningless and obsolete. This term fails to accurately distinguish absent (not present and nonrecoverable), latent (not present but can reemerge in the future), and covert (present but cannot be discerned by third-party observations) conscious awareness in the injured human brain. With improved understanding of human consciousness, it is more appropriate to use the term “disorders of consciousness,” because it indicates a continuum of phenotypes with variable degrees of conscious awareness (Gosseries et al. 2011). Neuroscience research further suggests that latent and covert awareness may persist in the dying human brain (Parnia et al. 2014; Rady and Verheijde 2016; Rouleau et al. 2016).

Clinical practice guidelines universally emphasize that brain death determination is a clinical diagnosis made by bedside clinical examination to confirm (1) unconsciousness or unresponsiveness, (2) absent cranial nerve motor reflexes, (3) absent spontaneous breathing (apnea), and (4) absent reversible causes (Shemie et al. 2006, 2014; The Academy of Medical Royal Colleges 2008; Wijdicks et al. 2010). Unconsciousness is assessed by absent behavioral motor response (e.g., eye opening and waking up) to auditory, tactile, and noxious stimuli. The question to be answered is: Do the tests recommended in the practice guidelines for brain death determination confirm the criteria of irreversible cessation of all functions of the entire brain including the brainstem? Indeed, several sources of scientific evidence have augmented concerns about the validity of clinical brain death determination.

First, neuroscience has established that latent and covert awareness can persist in the injured brain in the absence of behavioral motor responses to external stimuli, (Edlow et al. 2017; Fernández-Espejo et al. 2015). Clinical examination, a tool routinely used in the determination of brain death, is unreliable to assess the presence of latent and covert conscious awareness in this neurologic state when cognition may be dissociated from behavioral motor responses (Karakatsanis 2008). This neurologic state is similar to the “cognitive motor dissociation state” that has been described in patients with severe brain injuries (Schiff 2015, 2017). Therefore, clinical bedside examination alone can result in a misdiagnosis of absent capacity for conscious awareness in the severely injured human brain.

Second, the cranial nerves are part of the peripheral nervous system, transmitting afferent (sensory) signals to the central nervous system and efferent (motor) responses to skeletal muscles. These motor reflexes cannot be used to establish the presence or absence of higher integrative functions of the telencephalon (cerebral cortex), diencephalon, and limbic system (Karakatsanis 2008). In the clinical determination of brain death, it has been demonstrated that neuroendocrine, autonomic, and thermoregulatory functions may be present or recoverable after transient cessation (Joffe 2010; Nair-Collins et al. 2016; The President’s Council on Bioethics 2008).

Third, in brain death determination, an apnea test is performed to assess the absence of spontaneous breathing during the disconnection of mechanical ventilation. The apnea test is administered in order to ascertain irreversible injury to the respiratory centers in the medulla (part of the rhombencephalon or brainstem). However, 60% of brain dead patients with a positive apnea test were found to have a normal medulla at autopsy (Wijdicks and Pfeifer 2008). This finding is pertinent, since almost 50% of patients who were clinically determined brain dead continued to have both spontaneous movements (automatisms) and motor responses (reflexes) to noxious stimuli (Saposnik et al. 2009; Wijdicks et al. 2010). Some commentators have postulated that these spontaneous movements and motor responses to stimuli are mediated by the spinal cord because of its disconnection from the control of supraspinal centers in brain death (Termsarasab et al. 2015). Disconnection of the spinal cord from the supraspinal control will also result in a positive apnea test that is unrelated to the functional or structural integrity of the medullary respiratory centers. Infarction of the spinal cord at the junction with the medulla was reported in 42% of brain dead patients at autopsy (Walker 1978). Other confounding factors such as injury to the spinal cord, spinal nerves, and respiratory muscles can result in a positive apnea test in the presence of normal respiratory centers in the medulla (Joffe et al. 2010a, b). Failure to recognize these potential confounding factors can lead to a false-positive apnea test and, subsequently, a false determination of brain death. Additionally, hyperoxia and/or inadequate increase in carbon dioxide concentration in the arterial blood during the apnea test can also cause a false-positive apnea test and brain death diagnosis (Hansen and Joffe 2017; Joffe et al. 2010b; Shewmon 2017).

**Fig. 2:**
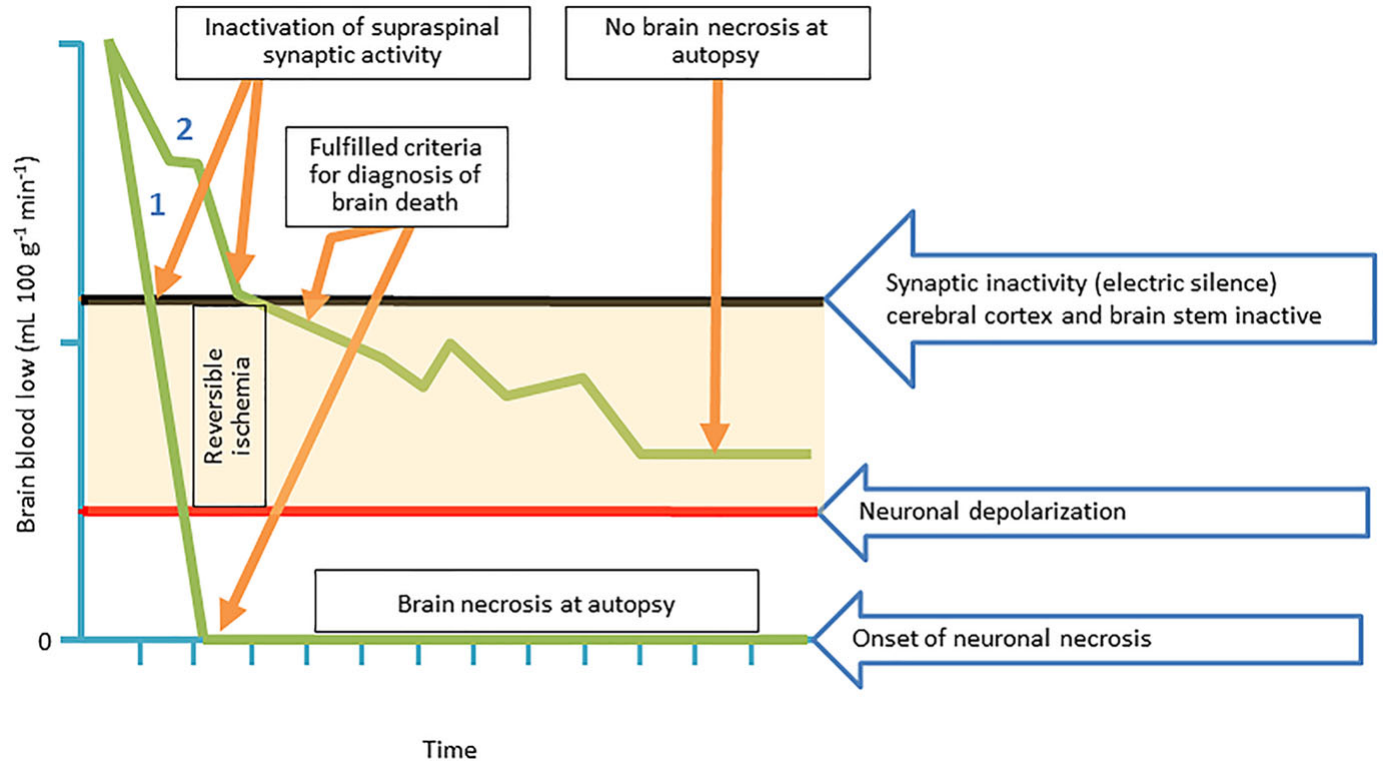
The phenomenon of global ischemic penumbra in the human brain. Acute reduction or cessation in whole-brain perfusion and blood flow has been postulated to be the final common pathophysiologic event in inducing brain death (Salih et al. 2016). The response of neurons in the human cerebral cortex to ischemia secondary to acute reduction and complete cessation of blood flow has been described (Dreier et al. 2018). The figure illustrates two distinct temporal patterns of whole-brain blood flow in two hypothetical patients (patient 1 and patient 2). With the initial reduction in brain blood flow, an early inactivation of supraspinal synaptic activity and suppression of the cerebral cortex and brainstem will result in developing the clinical criteria for the diagnosis of brain death (i.e., unresponsiveness, absent brainstem reflexes, and apnea) in both patients. Prolonged complete cessation of brain blood flow induces irreversible ischemia and neuronal depolarization in patient 1. Brain necrosis is observed at autopsy in patient 1, confirming the irreversibility of whole-brain ischemia. In patient 2, the acute reduction in blood flow induces reversible ischemia without triggering irreversible neuronal depolarization or the onset of necrosis (i.e., global ischemic penumbra). Autopsy in patient 2 demonstrates normal or minimal ischemia of brain structures and no evidence of necrosis. The temporal reversibility of the clinical findings of brain death associated with global ischemic penumbra in patient 2 is unknown. (Adapted from the original source by Coimbra (1999) in the Brazilian Journal of Medical and Biological Research 32: 1479–1487. The Brazilian Journal of Medical and Biological Research applies the Creative Commons Attribution License [CCAL] to all works published)

Fourth, the irreversibility of the above neurologic findings on clinical examination is determined by excluding other coexisting reversible conditions such as metabolic, endocrine, pharmacologic, or thermoregulatory perturbations that can suppress motor responsiveness, cranial nerve reflexes, or spontaneous breathing (Wijdicks et al. 2010). However, clinical determination of brain death ignores the most important factor for reversibility, which is the time from the initial brain injury to the potential recovery of conscious awareness. Salih et al. (2016) have demonstrated that acute reduction or cessation in whole-brain perfusion and blood flow is the final common pathophysiologic event in developing the clinical manifestations of brain death. However, only 40% of brain dead patients were found to have pathologic characteristics of ischemic brain necrosis (also called respirator brain) at autopsy (Walker 1978). It was also noted that about 10% of patients who were clinically determined brain dead had a normal brain at autopsy (Walker 1978). Patients who were clinically determined brain dead in accordance with the American Academy of Neurology practice guidelines had similar findings at autopsy (Wijdicks and Pfeifer 2008). The histopathologic examination identified normal or minimally ischemic changes in different regions of the telencephalon (the frontal lobe, temporal lobe, parietal lobe, occipital lobe, and basal ganglia) in about 40% of brain dead patients (Wijdicks and Pfeifer 2008). The thalamus, midbrain, pons, medulla, and cerebellum of brain dead patients at autopsy were found to be either normal or had minimal ischemic changes in 66, 63, 59, 60, and 48%, respectively (Wijdicks and Pfeifer 2008). The concern is then how to explain the absence of ischemic neuronal necrosis in patients who were clinically determined brain dead. Are the neurologic findings in these patients potentially reversible with time? Coimbra (1999) described the phenomenon of global ischemic penumbra to account for absent neuronal ischemia and brain necrosis in patients who were determined brain dead (Fig. 2). This phenomenon is characterized by a reduction in global blood flow to the whole brain that can result in suppression of supraspinal synaptic transmission in the cerebral cortex and brainstem without necessarily triggering irreversible ischemic neuronal damage. These patients would have the clinical manifestation of brain death, i.e., unresponsiveness, absent cranial nerve motor reflexes, and perhaps cerebral cortical electric silence, without the onset of irreversible neuronal depolarization and necrosis. At autopsy, these patients would also show no evidence of irreversible neuronal ischemia, necrosis, or brain damage. Dreier et al. (2018) have described the electrophysiologic responses of neurons in the dying human cerebral cortex to ischemia secondary to acute reduction and complete cessation of global blood flow. Indeed, their research confirmed that neuronal electric silence did not necessarily indicate irreversible neuronal ischemia (Dreier et al. 2018). This would further substantiate the existence of the phenomenon of global ischemic penumbra in some patients with the clinical diagnosis of brain death. Would these findings not indicate the potential for reversibility when sufficient time for recovery is allowed? Do these normal brain structures found at autopsy have the potential capacity to regain function? Indeed, isolated parts of the postmortem-fixed human brain (hippocampal body and parahippocampal gyrus) have been shown in tests to retain life-like functional responses to neurotransmitters applied in vitro (Rouleau et al. 2016). The hippocampus is part of the temporal lobe that has an important role in memory and awareness functions. The presence of normal brain structures in brain death should at least raise the possibility for reversibility of the capacity for consciousness in these patients. Additionally, neurogenesis has been documented in the dentate gyrus (part of the hippocampus and hippocampal formation) and in the striatum of the adult brain (Boldrini et al. 2018; Ernst et al. 2014; Gross 2000). Neuroplasticity can enable both the regeneration and the re-establishment of neural connectivity and restoring certain neurologic functions in the severely injured human brain (Sharma et al. 2013). The re-establishment of corticothalamic connectivity correlates with the recovery of conscious awareness (Di Perri et al. 2014). The role of neuroplasticity has been demonstrated in the recovery of consciousness and motor functions in the injured human brain (Huang et al. 2017; Thibaut et al. 2017). The preservation of these neuronal substrates in patients who are determined brain dead can play a role in the subsequent reemergence of consciousness after severe brain injuries. Electrophysiologic studies of the ischemic cerebral cortex and the postmortem-fixed human brain appear to substantiate that the reversibility timeline of neurologic findings in brain death has yet to be determined scientifically.

In summary, contrary to historical and current claims about the validity of the criteria and tests of determining brain death in clinical practice, contemporary neuroscientific findings indicate that some of its core assumptions are demonstrably incorrect, while others still lack sufficient evidential support (Table 1). If behavioral unresponsiveness does not equate to unconsciousness, then the current clinical practice of brain death determination is incongruent with empirical evidentiary findings. Neuroscience suggests that (1) motor reflexes cannot establish the presence or absence of higher integrative neurologic functions, (2) the sensitivity and specificity of the apnea test is questionable, and (3) the potential for recovery of conscious awareness through neurogenesis and neuroplasticity cannot be excluded. All these stand in sharp contrast to the frequently made assertion that (despite the fact that most practice guidelines have been assigned the weakest level of scientific evidence) the diagnosis of brain death can be made accurately on the basis of a comprehensive clinical examination alone (Wijdicks et al. 2010).

**Table 1:** Contemporary neuroscience challenges to clinical practice standards for brain death determination

Brain death determination*	Contemporary neuroscience challenges
Documentation on clinical examination Unresponsiveness (unconsciousness) Absent brainstem reflexes Positive apnea test Reversible causes must be excluded Confirmatory tests are only required when There is uncertainty about clinical examination An apnea test cannot be performed Cessation of functions of the whole brain are not required Neuronal necrosis at autopsy is not required to confirm the irreversibility of ceased brain functions	Bedside behavioral assessment of unresponsiveness does not necessarily equate with absent capacity for consciousness Cranial nerve motor (brainstem) reflexes cannot discern retained higher brain (telencephalon and diencephalon) functions Spontaneous movements and motor responses are postulated to be spinal automatisms and reflexes, respectively, because of spinal cord disconnection from supraspinal control, which also refutes the hypothesis that supraspinal (brainstem) control of respiration is being tested during the apnea test Situations when cessation of brain functions may not be irreversible There is the presence of normal brainstem and higher brain structures at autopsy Reduction in global blood flow to the whole brain can suppress supraspinal synaptic transmission and brainstem reflexes without triggering irreversible neuronal damage and necrosis (global ischemic penumbra) Living-like electrophysiologic responses of hippocampal body and parahippocampal gyrus of the fixed postmortem normal human brain are demonstrable in vitro Neurogenesis and neuroplasticity of the adult brain can regenerate and reestablish neuronal connectivity after injury

*The American Academy of Neurology Practice Parameter 2010 Evidence-based guideline update is the contemporary US standard in brain death determination (Wijdicks et al. 2010). Optional confirmatory tests may include electroencephalography, radioactive isotope cerebral perfusion scan, or cerebral vessel angiography. Contemporary neuroscience challenges also apply to other clinical practice standards for brain death determination such as the UK code of practice (The Academy of Medical Royal Colleges, 2008), the Canadian forum recommendations (Shemie et al. 2006), and the World Health Organization international guidelines (Shemie et al. 2014)

## Implications of Neuroscientific Findings on the Debate

### Philosophical and Legal Challenges

In addition to the philosophical concerns about the PCB’s rationale as outlined in a previous section, advances in neuroscience as described above have added an evidence-based element to the already philosophically muddy assumptions regarding the “irreversibility” of the cessation of all functions of the brain. In order to avoid ongoing controversies about the moment when a person has indeed died, Bernat (2013) has posited that the notion of “irreversibility” should be replaced by “permanence.” He has argued that this would be in line with the prevailing medical standard for death determination. “Permanence” is based on the notion that a ceased physiologic function *will not restart spontaneously* but can be restored through medical intervention. However, such intervention will not be performed in “permanence” (due to, for example, a do-not-resuscitate order). The term “irreversibility” connotes that the cessation of a physiologic function *can no longer be restored* through medical intervention (that is, such function is physiologically impossible to restore). From a biological perspective, an irreversibly ceased physiologic function is permanent but a permanently ceased physiologic function is *not* irreversible. Bernat has further posited that a death declaration at permanent cessation is routinely done without “a public outcry over it.” However, whether the public knows about this switch in the medical practice of death determination and understands its implications is questionable. From a biological perspective, permanent cessation of vital functions signifies the beginning of the dying process, whereas irreversible cessation signifies the completion of the dying process (Joffe et al. 2011). It has further been argued that the notion of permanence conflates prognosticating death with determining death (Byrne and Nilges 1993). If the chances for accurately prognosticating outcomes of care in individual cases have been widely considered rather poor, the emergence of neuroscientific findings in brain death determination appears to confirm that assessment.

More importantly, the paradigmatic change from the use of irreversibility to permanence represents a major deviation from both the PC and PCB’s commitment to a biological definition of death. Introducing a terminology change of this nature would require abandonment of the biological definition of death and replacing it with a non-biological definition. As stated in an earlier section, the latter qualifies as a hypothetical construct, i.e., one that can be debated philosophically but not one that can be proven or disproven by empirical methods. However, Bernat’s (2013) observation that death declaration at permanent cessation is routinely done appears to be substantiated by a survey study among neurologists, indicating considerable variances in the understanding of why brain death equates to human death. Neurologists’ reasoning for equivalence included congruence with the higher brain concept, with the concept of prognosis, and with the concept of the vital work of the organism, while 50% of those surveyed did not indicate support for any concept (Joffe et al. 2012). At least for this cohort of medical practitioners, Bernat’s observation appears to be correct. It would follow that death in the practice of medicine is defined on the basis of a non-biological definition, which is incongruent with the legal definition that, presumably, was formulated on the basis of an empirically verifiable biological definition. Noncompliance with the UDDA and determining death by non-biological criteria would also defeat the legally desired uniformity of death determination and raise the question why other such definitions of death should not also be afforded legitimacy. More importantly, not meeting the stipulation on the irreversibility standard formulated in the UDDA results in a false positive and a violation of the law (Nair-Collins et al. 2016). Citing Capron (1978), the PC stated: “[w]ere a non-uniform standard permitted, unfortunate and mischievous results are easily imaginable” (The President’s Commission 1981 p. 80). On top of the already existing controversies about the term “irreversibility,” the potential for and probability of covert awareness or capacity for recovery of consciousness presents an additional, evidence-based challenge to equating brain death to the biological death of a human being. On theoretical grounds, this strengthens the position that the philosophical underpinning of the definition of brain death is weak at best. From an ethics perspective, even the potential for residual awareness should invalidate the claim of equivalence. Within Western philosophical tradition, the potential for consciousness is broadly accepted as a minimal necessary condition for being considered a human person.

Neuroscientific evidentiary findings also create significant legal challenges to contemporary practice of brain death determination. Advocates of organ donation and transplantation practice have advanced alternative interpretations of the UDDA to legitimize the contemporary clinical practice standard in brain death determination and subsequent organ procurement from brain dead patients. Lewis et al. on behalf of the American Academy of Neurology Ethics, Law, and Humanities Committee stated:

The Uniform Determination of Death Act (UDDA), which states, ‘[a]n individual who has sustained either (1) irreversible cessation of circulatory and respiratory functions, or (2) irreversible cessation of all functions of the entire brain, including the brain stem, is dead. A determination of death must be made in accordance with accepted medical standards.’ When defining ‘accepted medical standards,’ *the authors of the UDDA chose not to specify clinical criteria* and instead declared that brain death must *be determined based upon standards ‘accepted by a substantial and reputable body of medical men and women as safe and efficacious* for the purpose for which [they are] being employed.’ (The President’s Commission 1981) The UDDA, *or a close approximation of it, has since been accepted as judicial or statutory law* in every state (Burkle et al. 2011) [emphasis added] (Lewis et al. 2018 p. 424).

Lewis et al. conflated the criteria with the tests in death determination. The authors of the UDDA assumed that the clinical and the legal criteria are identical (i.e., irreversible cessations of all functions of the entire brain, including the brainstem) and these criteria would not change over time. However, the authors anticipated that the tests underlying the “accepted medical standards” might evolve over time due to technological advances in medicine. These medical tests employed in the UDDA’s “accepted medical standards” must continue to confirm the congruence of the clinical and the legal criteria of death. Truog has, nevertheless, posited instead that permanent unconsciousness and apnea define the legal criteria of death. He stated: “The UDDA has served its purpose well. By drawing a bright line at the level of *permanent unconsciousness and ventilator dependence*, the UDDA has defined when a person should be considered dead, making it permissible for the person to be an organ donor if they wish” [emphasis added] (Truog 2018). This reinterpretation would invalidate contemporary practice guidelines for brain death determination as “acceptable medical standards.” These practice guidelines do not directly test or ascertain the irreversibility of cessation of the capacity for consciousness as outlined in the previous section. Furthermore, using the term “permanence” implies that unconsciousness and/or apnea can potentially be reversed with medical intervention. Because of the denial of medical intervention by foregoing life sustaining treatment or by procuring vital organs for transplantation, the practice guidelines become a self-fulfilling prophecy of irreversibility of brain death.

### Challenges to Organ Donation Practice

The inability of tests to accurately diagnose death is of particular concern in the circumstances of heart-beating organ procurement. The emerging neuroscientific questions regarding potential latent and covert awareness in brain dead individuals greatly increase the risk of violating the do-no-harm principle within the procurement practice itself. This is in addition to the harm of declaring a person dead based on flawed criteria and tests.

From a policy-making point of view, concern about residual awareness in donors is also likely to further jeopardize the public’s trust in the organ donation process. Organ procurement organizations (OPOs) already continuously describe it as “organ donation after death” without clarifying how death is being determined. One study has shown that OPO Web sites “predominantly provide positive reinforcement and promotional information rather than the transparent disclosure of organ donation process” (Woien et al. 2006). Then, the importance of informing the public about the organ donation process was even further marginalized by changing the donor registration language from “consent” to “authorization” (Iltis 2015). The Organ Procurement and Transplantation Network (OPTN) describes the term “authorization” as “[t]he act of granting permission for a specific act. This is sometimes called consent, which is not to be confused as informed consent” [https://optn.transplant.hrsa.gov/media/1200/optn_policies.pdf#nameddest=Policy_01. Accessed Nov 2, 2017]. The OPTN’s justification for the change was that the organ procurement process is not considered “medical,” and thus does not require informed consent. This position, however, may arguably only be logically maintained if a donor at the time of organ procurement is truly dead, a premise that is and has been at the heart of the debate.

### Challenges Regarding Abrahamic Faith Traditions’ Reliance on Concept Validity

The PC briefly presented the Jewish, Catholic, and Protestant theological doctrines. It posited that, from a Jewish perspective, the complete cessation of brain function would be synonymous with “physiological decapitation” and thus acceptable. Shewmon (2007) has convincingly argued against the biological equivalence of brain death and physiologic decapitation. Despite the biological flaw of the analogy of brain death with physiologic decapitation, some Jewish, Christian and Islamic scholars have accepted brain death as human death (Albar 2012; Ashkenazi et al. 2015; Fins 1995; Napier 2009; Tonti-Filippini 2012). Referring to the statement from Pope Pius XII that it “remains for the doctor to give a clear and precise definition of ‘death’ and the ‘moment of death’ of a patient who passes away in the state of unconsciousness” (Pope Pius XII, *The Prolongation of Life,* The Pope Speaks 4; 393, 396 [1957]),^1^ the PC argued that the theological position that the “human essence or soul departs at the moment of death is not inconsistent with the establishment, through neurological examination, of the time when death occurs” (The President’s Commission 1981 p. 11). In response to the theological formulation of death, in which death occurs at the moment the soul leaves the body, the PC further stated “[w]hether this happens when the patient loses psychological capacities, loses all brain functions, or at some other point, varies according to the teachings of each faith and according to particular interpretations of the scriptures recognized as authoritative” (The President’s Commission 1981 p. 42).

Some Roman Catholic writers, including Germain Grisez and Patrick Lee, appealing to principles derived from St. Thomas Aquinas, defend the original President’s Commission Report’s position that at least in principle “whole-brain death” is equivalent to human death (Lee and Grisez 2012 p. 282) due to the loss of the “radical capacity to sense” which is required for an organism to be a human one. However, as William May notes, they do not state whether whole-brain criteria can be diagnosed through current testing (May 2013). May, who is sympathetic to Grisez and Lee’s arguments, in the end believes that “the best scientific and scholarly studies demonstrate as false the claim that a human person is dead if the functioning of the entire brain, including the brain stem, is irretrievably lost” (May 2013 p. 309). May maintains that such a position does not imply any disloyalty to the teachings of the late Pope John Paul II on the determination of death.

Although much of the attention in this article has been on exploring the validity of the biological definition of death, it is of interest to note that reliance on the authority assigned within and mutually recognized by at least some of the entities representing both the biological and theological definitions of death can result in agreement on seemingly incommensurable perspectives on death. For instance, proponents of the biological definition recognize that they do not have the theological authority to determine death; nevertheless, they claim authoritative power to do so. Proponents of a theological definition, in turn, deny having medical authority but acknowledge they rely on the medical locus of authority to validate determining death or at least not invalidate the theological definition of death. A case in point is that Catholicism accepts the authority of medical science and its practitioners to determine with (presumably) scientific certainty that death has occurred. Moreover, it also asserts its theological authority in declaring that death determined by these criteria must be accepted as the definitive demarcation point of when the soul departs from the body. The Pontifical Academy of Sciences declared that “brain death is not a synonym for death, does not imply death, or is not equal to death, but ‘is’ death” (Battro et al. 2007) With both sides not challenging their respective domain of authoritative power, proponents of the biological definition gained moral support for an otherwise fallible definition. The Catholic Church implicitly accepted the biological definition of death to then merge it with its theological definition but, arguably, without consideration of the innate fallibility of science or reassessment of the current state of science. In doing so, the Catholic Church (and other Abrahamic faith traditions) did not need to weigh in on whether current organ transplantation practices are compatible with religious teachings. However, critics have pointed to three “critical weaknesses of the rationale, endorsed by most religions, for the justification of the concept of brain death. It assumes without validation that (1) the irreversible pathophysiologic conditions under which the soul would separate itself from the body have materialized in brain death, (2) the whole brain serves as the exclusive “integrator” of bodily functioning and is indeed the seat of the soul, and (3) the permanent loss of holistic integration and potencies is adequate proof of the soul’s absence from the body” (Verheijde and Potts 2010).

Religions, at least those based on Abrahamic faith traditions rely for their acceptance of brain death on the trust they have in medical experts to determine human death. Abrahamic faith traditions’ acceptability of the concept brain death is conditional upon: (1) equivalency with biological death, (2) clinical determination with scientifically verifiable criteria, and (3) alignment with the theological definition of death, i.e., the separation of the soul from the human body. Abrahamic faith traditions also mandate that death is determined with scientifically validated criteria that confirm *irreversibility* (rather than permanence) of cessation of biological functions (Inwald et al. 2000; Rady and Verheijde 2018). However, if death determination cannot be made with scientific certainty, the claim to authoritative power by medical practitioners in this regard appears undermined. Diminished authority in death determination implies reduced moral certainty. Both elements—the presence of scientific certainty and sufficient moral certainty—are prerequisites for religions to claim alignment of the biological definition of death with the theological definition of death. Emerging neuroscientific facts do not seem to have inspired religious leaders to reassess the validity of the current biological definition of death. Therefore, the question that remains unanswered is: If the procurement procedure is indeed the proximate cause of the donor’s death, can one still maintain that organ transplantation, although it may save lives, is morally permissible and compatible with religious teachings?

It is not surprising that several global religions including Abrahamic faith traditions continue to reject the medical conception and determination of brain death because of persistent scientific uncertainties (The Lancet 2011). The primary utility of declaring brain death in medical practice is to enable the procurement of transplantable vital organs without violating homicide laws in Western societies (Shah 2014). However, any religious tradition would reject theologically misaligned death determination, even if it is legally sanctioned, because the killing of donors for the purpose of procuring transplantable organs is universally forbidden. Several jurisdictions (such as New Jersey in the USA, Israel, and Japan) have permitted religious accommodation in death determination by legally proscribing neurologic determination of death if such a determination will “violate personal religious beliefs of an individual” in multicultural societies (Aramesh et al. 2018). In contrast, other jurisdictions (such as Nevada in the USA) have enforced nonconsensual brain death determination and potentially impeded personal rights to free exercise of religion (Rady and Verheijde 2018; Yanke et al. 2018). It is noteworthy that advocates for the legalization of the right to die by assisted suicide and euthanasia have also opposed introducing legislation to ratify religious exemption to brain death determination (Pope 2017). Legislative authorization of a medical conception of brain death that is grounded in a subjective assessment of neurologic disability deemed to be unacceptable for survival and without accommodation for valid moral and religious objections will transgress diverse values and individual liberty in multicultural societies.

## Conclusions

Contemporary neuroscientific findings appear to challenge the validity of death determination by neurologic criteria and to provide additional evidence supporting the rejection of the equivalency of brain death and biological death. If behavioral unresponsiveness does not equate to unconsciousness, then the clinical criteria and tests employed in brain death determination are incongruent with the philosophical underpinning of defining death with irreversible loss of the capacity for consciousness. Furthermore, if, as some have argued, the cessation of neurologic functions in clinical practice is routinely determined with the permanence standard rather than the irreversibility standard, then the true biological definition of death has already been abandoned in favor of a non-biological definition. Such silent transition is incongruent with the legal standard stipulated in the UDDA which was purportedly grounded in an empirically verifiable and uniform biological definition of death. The absence of a logically coherent philosophical concept of death and a valid set of clinical criteria and tests in brain death determination put the moral legitimacy of heart-beating organ procurement in jeopardy. Abrahamic faith traditions’ acceptability of the concept brain death was conditioned upon: (1) equivalency with biological death, (2) clinical determination with scientifically verifiable criteria, and (3) alignment with the theological definition of death, i.e., the separation of the soul from the human body. Brain death determination fails to meet these conditions. Therefore, new legislation should be enacted ratifying religious exemption to death determination by neurologic criteria. A paradigmatic change in death determination by neurologic criteria is necessary if transparency, trustworthiness, and moral and scientific integrity are to be preserved.

## Footnotes

1 In 2000, Pope John Paul II stated: “With regard to the parameters used today for ascertaining death—whether the ‘encephalic’ signs or the more traditional cardio-respiratory signs—the Church does not make technical decisions. She limits herself to the Gospel duty of comparing the data offered by medical science with the Christian understanding of the unity of the person, bringing out the similarities and the possible conflicts capable of endangering respect for human dignity.” [Address to 18th International Congress of the Transplantation Society August, 29 2000, §4. 39 Address to 18th International Congress of the Transplantation Society August, 29 2000, §5. http://w2.vatican.va/content/john-paul-ii/en/speeches/2000/jul-sep/documents/hf_jp-ii_spe_20000829_transplants.html (Accessed October 21, 2017)].
